# Efficient Hydrogenation of Xylose and Hemicellulosic Hydrolysate to Xylitol over Ni-Re Bimetallic Nanoparticle Catalyst

**DOI:** 10.3390/nano10010073

**Published:** 2019-12-30

**Authors:** Haian Xia, Lei Zhang, Hong Hu, Songlin Zuo, Li Yang

**Affiliations:** 1 Jiangsu Provincial Key Lab for the Chemistry and Utilization of Agro-Forest Biomass, College of Chemical Engineering, Nanjing Forestry University, Nanjing 210037, China; lzhang1117@163.com (L.Z.); honghu@njfu.edu.cn (H.H.); zuosl@hotmail.com (S.Z.); 2 Jiangsu Co-Innovation Center of Efficient Processing and Utilization of Forest Resources, Nanjing Forestry University, Nanjing 210037, China

**Keywords:** xylose, xylitol, Ni-Re alloy, hydrogenation, electron transfer

## Abstract

A disadvantage of the commercial Raney Ni is that the Ni active sites are prone to leaching and deactivation in the hydrogenation of xylose to xylitol. To explore a more stable and robust catalyst, activated carbon (AC) supported Ni-Re bimetallic catalysts (Ni-Re/AC) were synthesized and used to hydrogenate xylose and hemicellulosic hydrolysate into xylitol under mild reaction conditions. In contrast to the monometallic Ni/AC catalyst, bimetallic Ni-Re/AC exhibited better catalytic performances in the hydrogenation of xylose to xylitol. A high xylitol yield up to 98% was achieved over Ni-Re/AC (n_Ni_:n_Re_ = 1:1) at 140 °C for 1 h. In addition, these bimetallic catalysts also had superior hydrogenation performance in the conversion of the hydrolysate derived from the hydrolysis reaction of the hemicellulose of *Camellia oleifera* shell. The characterization results showed that the addition of Re led to the formation of Ni-Re alloy and improved the dispersion of Ni active sites. The recycled experimental results revealed that the monometallic Ni and the bimetallic Ni-Re catalysts tended to deactivate, but the introduction of Re was able to remarkably improve the catalyst’s stability and reduce the Ni leaching during the hydrogenation reaction.

## 1. Introduction

The depletion of fossil resources has attracted considerable interest in the valorization of renewable biomass materials for the production of valuable chemicals. The catalytic conversion of naturally occurring sugars, such as xylose and glucose, into their corresponding sugar alcohols is an attractive pathway for the production of value added chemicals, such as sweeteners and pharmaceutical intermediates [1,2,3,4,5,6,7,8,9,10,11]. Xylitol is an important artificial sweetener and has been widely used in various fields [12,13,14,15,16]. At present, the production of xylitol proceeds mainly through the reduction reaction of xylose derived from the hydrolysis of corncob [17,18,19,20,21,22]. *Camellia oleifera* is a woody oil crop that is distributed in the southern provinces of China [23,24,25,26]. The seeds of *camellia oleifera* are used to produce an edible oil, which contains plentiful vitamins and unsaturated fatty acids and is comparable to the well known olive oil. However, *Camellia oleifera* shell, generated from the process residues, is usually neglected and still not used efficiently. More than three million tons of *Camellia oleifera* shell are annually produced as agricultural waste, and most of this is burned to provide thermal power [25]. *Camellia oleifera* shell is a lignocellulosic biomass that is composed of a high content of hemicellulose and lignin and a low content of cellulose. Therefore, *Camellia oleifera* shell is considered as an alternative raw material to corncob for the production of xylose and xylitol due to its high hemicellulose content.

**Scheme 1:**
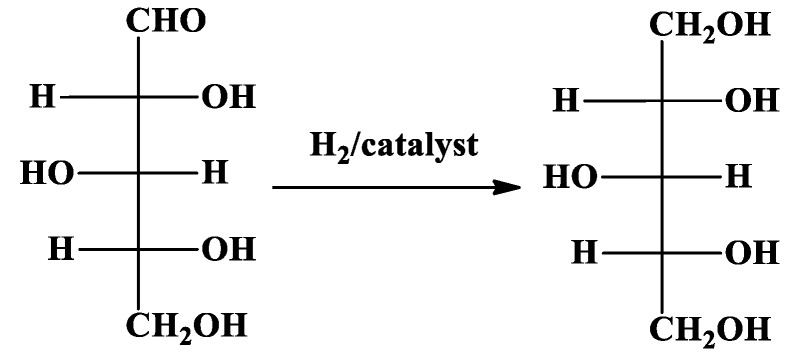
Hydrogenation of xylose to xylitol.

The catalytic hydrogenation of xylose into xylitol is traditionally performed in a three phase slurry batch reactor on Raney Ni catalysts (Scheme 1) [12,27]. The Raney Ni catalyst exhibited high activity and excellent xylitol yield with corn stover hydrolysate as a starting material [28]. The main advantages of Raney Ni catalyst were its low price, good activity, and high selectivity. However, a major disadvantage of Raney Ni catalysts is their easy deactivation due to Ni metal leaching and the deposition of the reaction byproducts on the surface active Ni sites [14,27,29,30]. Moreover, Ni ions leached into xylitol solution must be removed when xylitol is used in the food industry or as medicine, which leads to an increase in the production cost of xylitol because of expensive purification steps. In recent years, much effort has been undertaken to develop more stable and recyclable catalysts for the hydrogenation of xylose into xylitol [28,31,32,33]. Pecchi et al. successfully prepared Ni containing perovskite-type oxides, such as La_1−*x*_Ce*_x_*Al_0.18_Ni_0.82_O_3_ (*x* = 0.0, 0.1, 0.5, 0.7) and Nd_1−*x*_Ce*_x_*Al_0.16_2Ni_0.838_O_3_ (*x* = 0.0, 0.1, 0.5, 0.7) catalysts, for the hydrogenation of xylose to xylitol [30,31]. In their work, the reaction products were composed of xylitol, xylulose, glycerol, and ethylene glycol, and selectivity toward xylitol up to 50% was obtained at 100 °C under 25 bar H_2_ pressure [30]. Moreover, it was found that the formation of crystalline structures rendered substantial leaching resistant catalysts because no significant Ni leaching was detected in aqueous medium for these catalysts with Ce-free, as well as 50% and 70% Ce contents [30]. It is still a great challenge to develop novel catalysts with highly stable and excellent xylitol selectivity in the catalytic hydrogenation of xylose under mild conditions. 

In addition to the Ni based catalysts, various noble metals, such as Pt, Pd, and Ru, have been utilized for the catalytic hydrogenation of xylose to xylitol [29,34,35,36]. It has been reported that Ru based catalysts exhibit good catalytic performance and no leaching occurred during the xylose hydrogenation reaction [14,36], which is an alternative catalyst to Raney Ni. For example, Hemandez-Mejia et al. reported that Ru supported on rutile titania afforded 100% xylose conversion and up to 98% xylose yield at 120 °C in 15 min [36]. Nevertheless, in comparison with the Ni based catalyst, the use of a noble metal catalyst is very expensive and not the most desirable choice for a large scale xylose hydrogenation process. 

It has been revealed that the application of bimetallic catalysts could remarkably boost the catalytic activity and improve the stability [37,38,39,40]. Zhu et al. reported that the bimetallic Ni-Re catalysts had better hydrodeoxygenation performances of phenol into benzene compared to the monometallic Ni catalyst due to the geometric effect and electronic effect after the introduction of Re metal [38]. However, Mikkola et al. revealed that a Mo promoted Raney Ni catalyst was prone to deactivation due to the collapse of the catalyst pore structure and leaching of the promoter metals, Mo and alumina [27]. It has been widely reported that most metal oxide supports are unstable and prone to undergo structural destruction or component leaching in aqueous hydrogenation reactions. AC has been shown to have a good resistance to acids and bases, and it contains a variety of surface oxygen-containing functional groups which can strongly anchor the active metal sites. Thus, AC is an excellent support for most metal nanoparticle catalysts [41]. Therefore, it is of great significance for the development of a more stable and efficient bimetallic Ni-based catalyst in the catalytic hydrogenation of xylose. 

In this work, activated carbon (AC) supported Ni-Re bimetallic catalysts with various Ni/Re ratios were synthesized via the impregnation method. The aim of the use of supported catalysts is to decrease the Ni loading and enhance its mechanistic intensity, thereby decreasing the catalyst cost. The incorporation of Re into N based catalysts is to enhance its catalytic activity, as well as improve its hydrothermal stability and decrease Ni leaching. The catalytic performances of these catalysts were evaluated by the hydrogenation of xylose and hydrolysate containing xylose derived from the hydrolysis of *Camellia oleifera* shell. The physicochemical properties of various catalysts were characterized by X-ray photoelectron spectroscopy (XPS), X-ray diffraction (XRD), CO chemisorption, and transmission electron microscope (TEM) techniques. The deactivated mechanism of these catalysts was also discussed. 

## 2. Materials and Methods 

### 2.1. Catalyst Preparation

The monometallic Ni/AC or Re/AC and the bimetallic Ni-Re/AC catalysts with different Ni/Re ratios were synthesized by the wet impregnation method. The activated carbon was derived from coconut husk produced by steaming activation (S_BET_ = 1500 m^2^/g). Ni(NO_3_)_2_.6H_2_O and NH_4_ReO_4_ were used for the catalyst precursors. A typical synthesis procedure is described as follows. First, desired amounts of Ni(NO_3_)_2_.6H_2_O and NH_4_ReO_4_ were dissolved into deionized water, and then, the activated carbon was added into the aforementioned aqueous solution. Subsequently, the mixture was stirred for 12 h, followed by removing water using a rotary evaporator. Finally, the resulted solid was calcined at 500 °C in N_2_ flow for 2 h and then was reduced in 1 vol%H_2_/N_2_ flow at the same temperature for 2 h. The Ni loading of the catalysts was fixed to 5 wt%, and the Ni/Re ratios were 10:1, 4:1, 2:1, and 1:1. The catalysts were denoted as: Ni-Re/AC(n_Ni_:n_Re_ = x:y), where n_Ni_:n_Re_ refers to the Ni/Re molar ratio. For comparison, a Re/AC with Re content of 15.8% was also fabricated, and its Re content was equal to that of Ni-Re/AC(n_Ni_:n_Re_ = 1:1). 

### 2.2. Catalyst Characterization

The XRD patterns were recorded on a Rigaku Ultima IV X-ray diffractometer (Rigaku, Matsubaracho, Japan) equipped with a Cu Kα X-ray source operating at 40 kV and 30 mA. The TEM images were measured on a JEM-200CX transmission electron microscope (JEOL, Tokyo, Japan). The size of every particle was obtained through measuring its spherical diameter by the manual approach, and then, a mean particle size was calculated by averaging the diameters of ~300 particles. The XPS analyses were performed using a KRATOS AXIS Ultra DLD instrument (Shimadzu, Tokyo, Japan). An Al Kα X-ray source was used for all samples, along with pressure in the analysis chamber of 7 × 10^−8^ Pa. The step size of 1 eV and the dwell time of 0.1 s were employed, and each peak was scanned once for the survey scans. 

The Ni dispersion was measured by CO chemisorption on an AutoChem II 2920 instrument (Micromeritics, Norcross, GA, USA). Prior to CO chemisorption, the samples were reduced at 500 °C for 1 h in flowing H_2_, followed by He purging for another 30 min. Subsequently, the sample was cooled to 50 °C, and then, 10% CO/He was pulsed into the reactor through the six port valve until a constant CO peak area was reached. 

### 2.3. The Preparation and Purification of Hydrolysate

The hydrolysates were obtained by dilute sulfuric acid treatment of crushed *Camellia oleifera* shell, and the reaction results can be seen in Table S1 of the Supplementary Material. The liquid fraction was separated from the solid fraction by vacuum filtration. After the hydrolysis, the pH value of the hydrolyte was adjusted to ca. 5 using CaCO_3_, and the resulting gypsum was removed by filtration through vacuum filtration. After the neutralization, the resulting solution was further purified by activated carbon and then ion exchanged resin. Finally, the purified solution was evaporated and concentrated to a desired concentration. 

### 2.4. Catalytic Reaction

In a typical procedure, the catalyst (0.10 g), xylose or hydrolysates, and deionized water (50 mL) were added into a 100 mL stainless steel reactor. The reactor was purified with pure N_2_ and H_2_ for three cycles, respectively, and finally pressurized with 2 MPa of H_2_ at room temperature. The autoclave was heated to a desired reaction temperature and kept at this temperature for 2 h under stirring. After the reaction, the autoclave was cooled to room temperature before the liquid solution was separated from solid catalyst by filtration. In each experiment, three parallel experiments were conducted, and the error was obtained within 5%.

The liquid product was identified on an Agilent 1200 S LC (Agilent, Palo Alto, CA, USA) with a NH_2_-S column with a refractive index detector (RID) by using a mixture of acetonitrile and water (75:25) as a mobile phase at a flow rate of 0.8 mL/min at 303 K. The residual solid was washed with deionized water several times followed by drying at 333 K overnight. The conversion of xylose was determined. The xylose conversion and yields of xylitol were calculated according to Equations (1) and (2):

Conversion (%) = (xylose mole input in the reactor − xylose mole after the reaction)/xylose input in the reactor × 100(1)

Yield (%) = xylitol mole after the reaction/xylose mole input in the reaction × 100(2)

The stability of the catalysts was tested for two or three successive runs. The catalysts after reaction were washed with deionized water several times before the next run. For the catalyst regeneration procedure, the recovered solid was firstly dried and then further reduced in 1 vol% H_2_/N_2_ flow at 500 °C for 2 h. The leaching percentage of Ni in the filtrate obtained from the first run reaction was determined by ICP-OES. 

## 3. Results and Discussion

### 3.1. XRD Results

**Figure 1:**
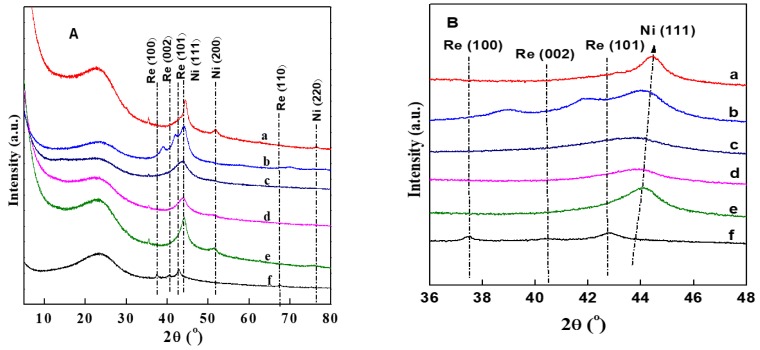
XRD patterns of (**A**) (a) Ni/activated carbon (AC), (b) Ni-Re/AC(n_Ni_:n_Re_ = 1:1), (c) Ni-Re/AC(n_Ni_:n_Re_ = 2:1), (d) Ni-Re/AC(n_Ni_:n_Re_ = 4:1), (e) Ni-Re/AC(n_Ni_:n_Re_ = 10:1), and (f) Re/AC. (**B**) Magnification of (A).

The XRD was used to study the crystalline structure of the monometallic Ni/AC, Re/AC, and bimetallic Ni-Re/AC catalysts. As shown in Figure 1A, for 5% Ni/AC, several diffraction peaks were observed at 22.5°, 44.5°, 51.7°, and 76.4°. The peak at 22.5° was assigned to the activated carbon support, whereas the three peaks at 44.5°, 51.7°, and 76.4° were ascribed to Ni (111), Ni (200), and Ni (220) of face centered cubic (FCC) Ni nanoparticles [42,43], respectively. For the monometallic Re/AC (15.8 wt%) catalyst (Figure 1(f)), four diffraction peaks appeared at 37.5°, 40.6°, 42.6°, and 67.1°, which were attributed to Re (100), Re (002), Re (101), and Re (110) of hexagonal closed packed (HCP) Re metal [38], respectively. After the incorporation of Re species, the signal assigned to Ni (111) shifted to a small diffraction angle. With increasing Re content, besides the diffraction peaks related to Ni nanoparticles, two new signals at 38.8° and 42.0° were observed for the Ni-Re/AC (n_Ni_:n_Re_ = 1:1) catalyst. Moreover, the diffraction peak related to Re (100) at 2θ of 37.5° shifted to a bigger value at 38.8°, and simultaneously, the signals assigned to Re (002), and Re (101) shifted to higher values (Figure 1B). The shift in their diffraction angles demonstrated that the lattice expansion of the Ni unit cell took place, likely due to the incorporation of Re into the Ni lattice during high temperature reduction. The results clearly suggested the formation of Ni-Re alloy in the bimetallic Ni-Re catalyst. In addition, there were no diffraction peaks ascribed to ReO_x_ species in the monometallic Re/AC or the bimetallic Ni-Re catalysts, indicating that ReO_x_ species could be highly dispersed on all Ni-Re/AC samples. 

### 3.2. TEM Results

**Figure 2:**
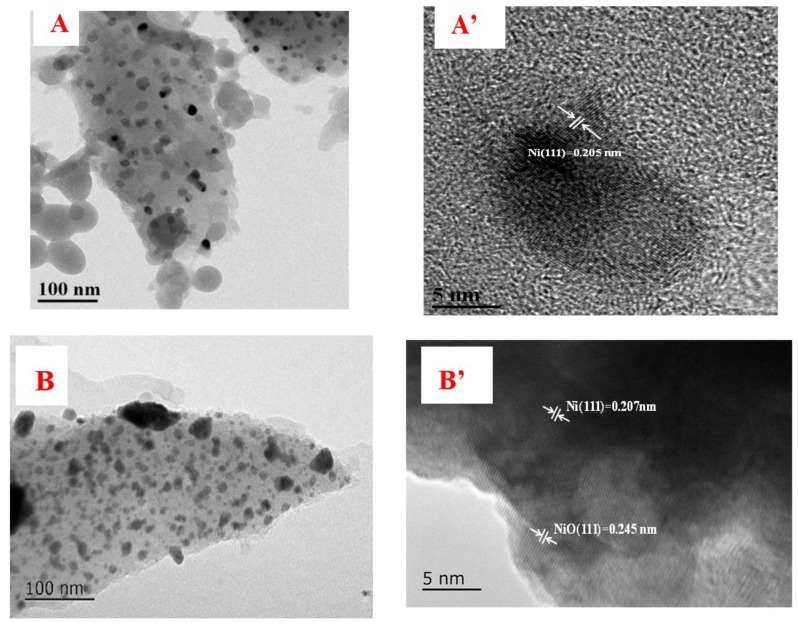

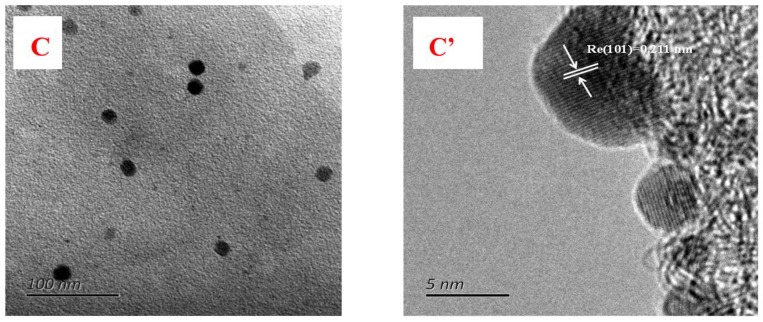
Three representative TEM images and their HRTEM images of Ni/AC (**A**,**A’**), Ni-Re/AC (n_Ni_:n_Re_ = 1:1) (**B**,**B’**), and Re/AC (**C**,**C’**).

Figure 2 shows the three representative TEM and their high resolution TEM (HRTEM) images of the Ni/AC and Ni-Re catalysts, and the corresponding mean Ni particle sizes are listed in Table 1. It can be seen from Figure 2A–C that Ni particles or Re oxide particles were highly dispersed and uniformly distributed on the AC support surface. 

**Figure 3:**
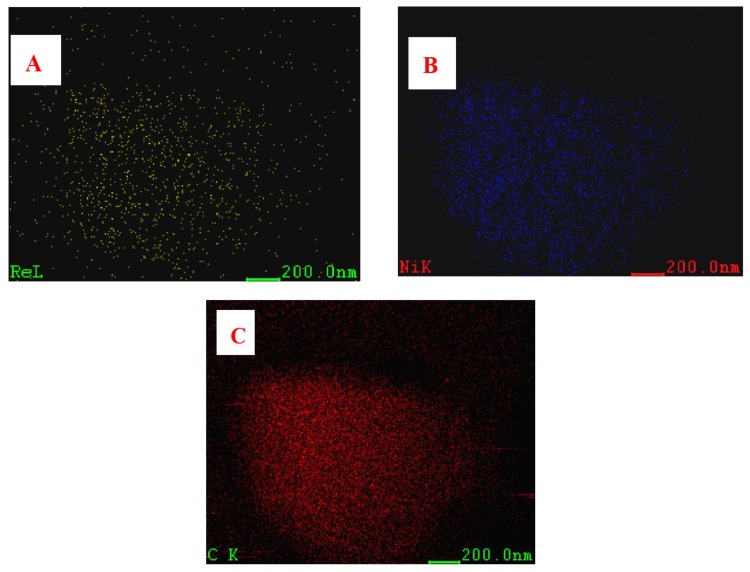
The elemental mapping of 5% Ni-Re/AC (n_Ni:_n_Re_ = 1:1) catalyst: (**A**) Re; (**B**) Ni; and (**C**) C.

Table 1 shows that a large particle size of ca. 22.0 nm was observed for the monometallic Ni/AC catalyst. After the introduction of Re, the particle size became smaller and more uniform compared to the Ni/AC catalyst. Moreover, with an increase of Re metal, the average particle size decreased from ca. 22.0 nm for Ni/AC to ca. 13.0 nm for Ni-Re/AC (n_Ni_:n_Re_ = 1:1), showing that the addition of Re can effectively promote the dispersion of Ni metal nanoparticles (Table 1, Entries 2 through 6). The possible interpretation for the observation was that the introduction of Re was mostly like a baffle plate, which could prevent the further migration and agglomeration of Ni atoms or small particles from clustering into larger sized particles by the formation of alloy during the high temperature reduction process. HRTEM images were also taken to investigate the fine structures (Figure 2A’–C’). For the monometallic Ni/AC catalyst, the resulting lattice fringe was approximately 0.205 nm, which was attributed to that of Ni (111). After the incorporation of Re species, the lattice fringe was approximately 0.207 nm, which was greater than that of Ni (111) for Ni/AC, but smaller than that of Re (101) (0.211 nm) for Re/AC. The results were also consistent with the XRD results that the penetration of Re atoms into the Ni lattice during H_2_ high temperature reduction led to the expansion of the lattice, which further showed that the Ni-Re alloy was formed after the incorporation of Re species. The composition of the bimetallic Ni-Re/AC (n_Ni_:n_Re_ = 1:1) catalyst was further analyzed by the EDS mapping method (Figure 3). As can be seen, the Ni and Re elements were uniformly distributed on the AC support, which was also further indicative of the formation of Ni-Re alloy in these bimetallic catalysts.

### 3.3. XPS Results

**Figure 4:**
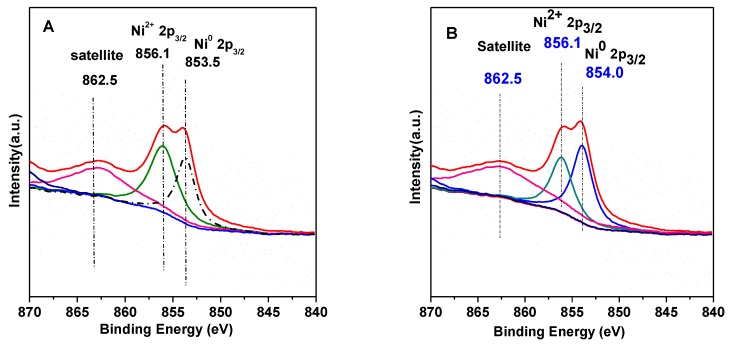
Two representative Ni 2p XPS of (**A**) 5% Ni/AC and (**B**) Ni-Re/AC(n_Ni_:n_Re_ = 1:1).

XPS was used to analyze the surface elemental composition and valence state of the catalyst. The XPS results of Ni 2p regions for Ni/AC and the bimetallic Ni-Re catalysts are illustrated in Figure 4. As shown, the two samples exhibited similar Ni 2p spectra. Besides a binding energy at approximately 862.5 eV assigned to the shake-up satellite of Ni 2p_3/2_, two peaks at approximately 853.0 and approximately 856.0 eV were observed, which were ascribed to Ni^0^ and NiO species [38,44], respectively. Moreover, it was observed that the binding energy of Ni^0^ species shifted from 853.5 eV for Ni/AC to a higher binding energy of 854.0 eV for Ni-Re/AC(n_Ni_:n_Re_ = 1:1). Similar observations were also found for other Ni-Re samples (see Figure S2 in the Supplementary Material). 

**Figure 5:**
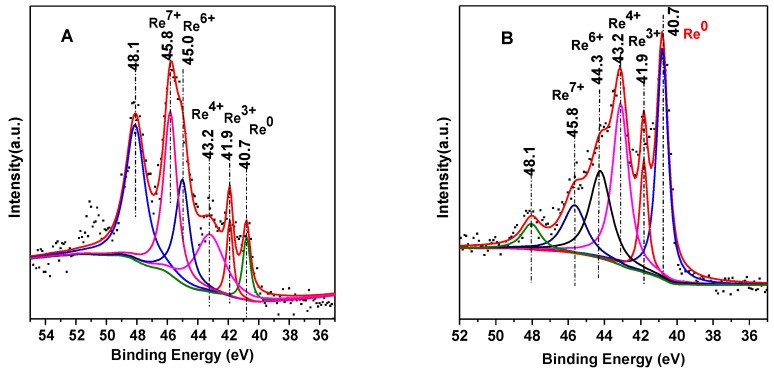
Two representative Re 4f XPS of (**A**) 15.8% Re/AC and (**B**) Ni-Re/AC (n_Ni_:n_Re_ = 1:1).

Figure 5 presents the XPS spectra of the Re 4f region for Re/AC and Ni-Re/AC(n_Ni_:n_Re_ = 1:1). For the two samples, five deconvoluted peaks appeared at 40.8, 41.9, 43.2, 45.0, and 45.8 eV, which were assigned to the 4f_7/2_ of Re^0^, Re^3+^, Re^4+^, Re^6+^, and Re^7+^ species [38,44], respectively. Similar phenomena for Re 4f spectra were also found for other Ni-Re samples (See Figure S3 in the Supplementary Material). The presence of different oxidation states was attributed to the strong oxophilicity of Re. It should be noted that partial oxidation of Re cannot be avoided upon exposure to air. Furthermore, it was observed that the peak intensities of Re^0^ together with Re^3+^ and Re^4+^ species of Ni-Re/AC(n_Ni_:n_Re_ = 1:1) were higher than those of Re/AC, albeit the two samples contained the same Re loading, while the situation was converse for Re^6+^ and Re^7+^ with high valence states for the two samples. The results clearly suggested that the Re species were reduced readily to lower valence states or low valence state species (Re^0^, etc.) were more difficult to oxidize due to the formation of the Ni-Re alloy. 

### 3.4. CO Chemisorption

The amount of surface active Ni sites was determined by CO chemisorption. For the Re/AC catalyst, the amount of adsorbed CO could be neglected, suggesting that Re species did not adsorb CO at a low temperature. Thus, CO adsorption on the bimetallic Ni-Re catalysts may be regarded as only related to the active Ni sites. As shown in Table 1, CO/Ni increased from 0.02 for Ni/AC to 0.08 for Ni-Re/AC(n_Ni_:n_Re_ = 1:1) and 0.027 for Ni-Re/AC(n_Ni_:n_Re_ = 2:1). For Re/AC, no CO adsorption was detected. These results also showed that the incorporation of Re species markedly enhanced the formation of surface active Ni sites. 

### 3.5. Catalytic Performances

The effect of reaction temperature on the conversion of xylose and the yield of xylitol was investigated (Table 2). It can be inferred from Table 2 that a xylitol yield of 77.6% with a xylose conversion of 79.0% was obtained over Ni-Re/AC (n_Ni_:n_Re_ = 1:1) at 120 °C for 2 h. As the reaction temperature was greater than 140 °C, complete conversion of xylose was achieved, but the xylitol yield was reduced from 95.6% at 140 °C to 64.8% at 200 °C (Table 2, Entries 4 and 7). This was due to the fact that high temperature led to the formation of byproducts including furfural produced from xylose dehydration, even ethylene glycol (EG), and 1,2-propanediol formed from xylitol hydrogenolysis at 200 °C.

The influence of the reaction time on the hydrogenation of xylose to xylitol was also investigated. When the reaction time was 0.5 h, a xylitol yield of 57.2% at a xylose conversion of 59% was afforded over Ni-Re/AC (n_Ni_:n_Re_ = 1:1) at 140 °C. A high yield up to 98.0% was achieved at 140 °C for 1 h. As the reaction time was extended to 2 h, the xylitol yield slightly dropped to 95.6% because of the occurrence of side reactions such as xylose dehydration.

**Figure 6:**
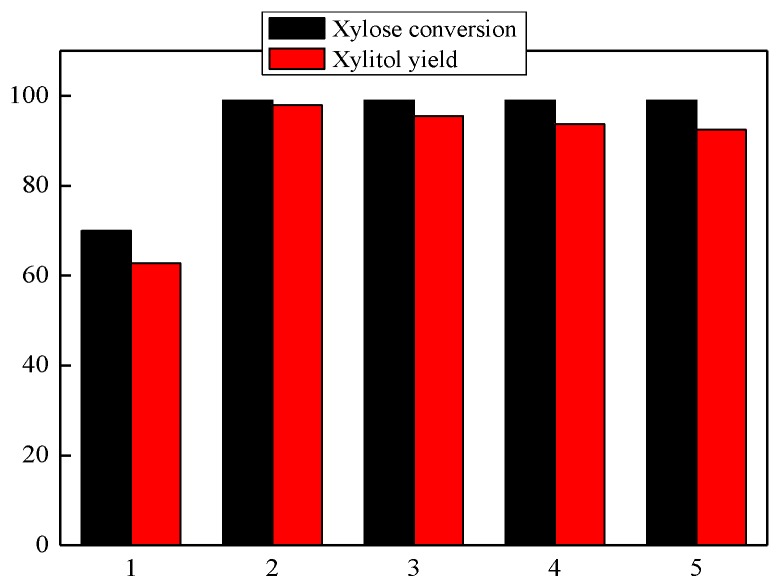
Effect of the Ni/Re ratio on the conversion of xylose and the yield of xylitol over various catalysts: (1) Ni/AC; (2) Ni-Re/AC (n_Ni_:n_Re_ = 1:1); (3) Ni-Re/AC (n_Ni_:n_Re_ = 2:1); (4) Ni-Re/AC (n_Ni_:n_Re_ = 4:1); (5) Ni-Re/AC (n_Ni_:n_Re_ = 10:1). Reaction conditions: 1.0 g xylose, 20.0 mL deionized water, 0.1 g catalyst, 2 MPa of H_2_, and 140 °C for 1 h.

Figure 6 shows the effect of the Ni/Re ratio on the hydrogenation of xylose to xylitol over various catalysts at 140 °C for 1 h. Ni/AC afforded a xylitol yield of 62.7% with a xylose conversion of 70%. As the Re loading increased, complete xylose conversion was achieved over four Ni-Re bimetallic catalysts, suggesting that the incorporation of Re could obviously promote the xylose conversion, i.e., the formation of alloy favored the conversion of xylose and enhanced the hydrogenation activity. Additionally, an increase in Re content resulted in an increase in xylitol yield from 92.5% for Ni-Re/AC (n_Ni_:n_Re_ = 10:1) to 98% for Ni-Re/AC (n_Ni_:n_Re_ = 1:1). The possible explanation is that as the Re loading increased, the particle size also decreased, resulting in approximately 16.0 nm for Ni-Re/AC (n_Ni_:n_Re_ = 10:1) to approximately 13.0 nm for Ni-Re/AC (n_Ni_:n_Re_ = 1:1) because smaller particle sizes afforded higher catalytic activity. It should be noted that the monometallic Re/AC catalyst did not show any hydrogenation activity of xylose under identical reaction conditions.

**Figure 7:**
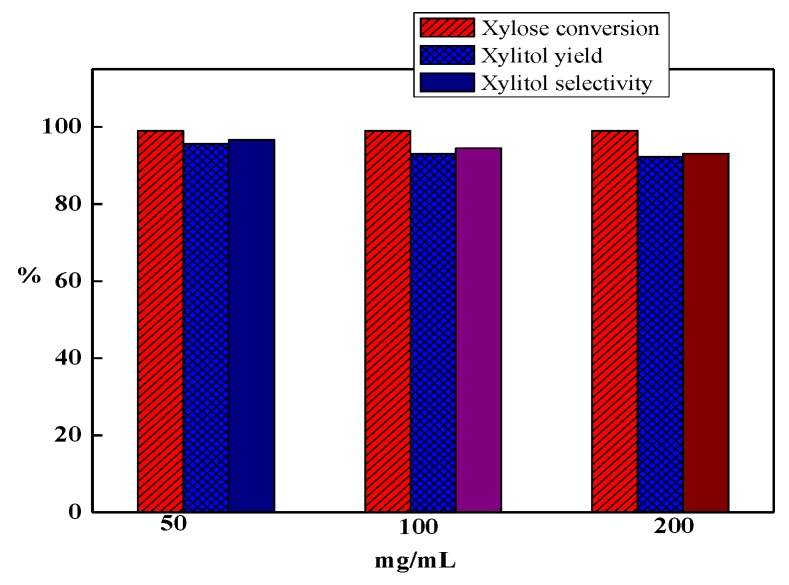
Effect of xylose concentration on the conversion of xylose, the xylitol yield, and the selectivity toward xylitol over Ni-Re/AC (n_Ni_:n_Re_ = 1:1). Reaction conditions: 20 mL deionized water, 0.1 g catalyst, 2 MPa of H_2_, and 140 °C for 1 h.

Figure 7 displays the impact of xylose concentration on the hydrogenation of xylose to xylitol on Ni-Re/AC (n_Ni_:n_Re_ = 1:1). When the xylose concentration was 50 mg/mL, a xylitol yield of 96% was obtained. As the xylose concentration further increased, the xylitol yield was only slightly reduced to 93% for a 100 mg/mL concentration and 92% for a 200 mg/mL concentration. This also indicated that the catalyst still kept its high catalytic performances even as the xylose concentration was close to 20%. 

Table 3 summarizes the experimental conditions and the catalytic results of the hydrogenation of xylose to xylitol. Most of the noble metal catalysts (i.e., Pd, Ru, Rh) and non-noble metal catalysts (i.e., Ni) were active for the xylose hydrogenation. A comparison of the catalytic properties reported in the literature is very complicated because of various experimental conditions. It can be inferred from Table 3 that the Ru/AC catalyst gave a high selectivity up to 98.7% toward xylitol. Moreover, it was found that AC was an excellent support because Ru/AC showed a superior catalytic performance as compared to other supported Ru based catalysts. The Ru based catalysts were demonstrated to have a good stability in catalyst recycling experiments. The disadvantage of the Ru based catalysts was that Ru metal was more expensive than Ni metal. Ni-Re/AC used in this work exhibited a comparable performance to that of Raney Ni or Ru/TiO_2_. The drawback of Ni based catalysts was that the surface Ni active sites were prone to leaching.

### 3.6. Hemicellulosic Hydrolysate Hydrogenation Reaction

The hemicellulosic hydrolysate was also used for the hydrogenation of xylose into xylitol. The authors employed the optimal reaction conditions that were obtained by optimizing the reaction conditions using commercial xylose as the model substrate. The obtained hydrolysate generated from the hydrolysis reaction of hemicellulose of *Camellia oleifera* shell was used for xylose hydrogenation reaction at 140 °C for 1 h. A high xylose yield of 88.2% with a xylose conversion of 93.2% was achieved under the optimized reaction conditions. An inferior yield of xylose using the hydrolysate as the starting material was obtained in comparison with using commercial xylose because the hydrolysate contained a higher concentration of metal impurity ions or other trace impurities, such as lignin fragments, which led to a reduction in the hydrogenation activity of xylose.

### 3.7. The Recycling Experiment

**Figure 8:**
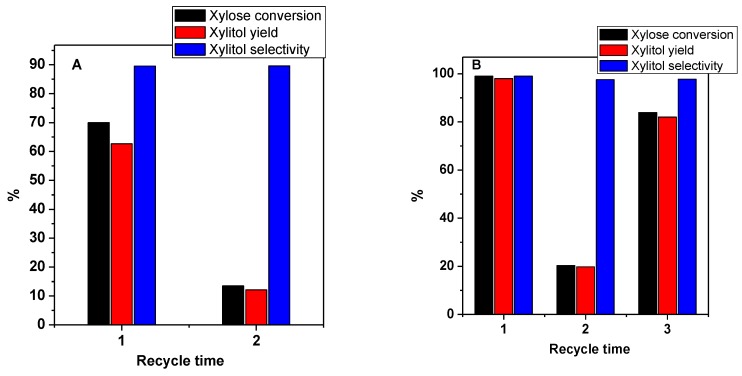
Reusability of (**A**) Ni/AC and (**B**) Ni-Re/AC(n_Ni_:n_Re_ = 1:1) catalysts. Reaction conditions: 20 mL deionized water, 0.1 g catalyst, 2 MPa of H_2_, and 140 °C for 1 h.

The long term stability of heterogeneous catalyst is very important for catalytic reactions. Thus, the stability of the catalysts (Ni/AC and (b) Ni-Re/AC(n_Ni_:n_Re_ = 1:1)) catalysts) in the hydrogenation reaction of xylose was evaluated. The recovered catalysts were washed and dried before their reuse. As shown in Figure 8, for the second run, the xylose conversion and xylitol yield were substantially reduced, but the xylitol selectivity was not changed, showing that the two catalysts were very prone to deactivation. According to the literature results previously mentioned in the Introduction, the authors prospected that the catalyst deactivation was owed to the deposition of organic compounds on the active Ni sites. Thus, a regeneration procedure was employed under 1% H_2_ flow at 500 °C for 2 h. After the regeneration, the third recycle was conducted. The result showed that the catalyst’s activity was markedly recovered, but the xylose conversion and xylitol yield were still lower than those obtained from the fresh catalyst. The deactivation reason was further analyzed by ICP-OES, and the results revealed that the Ni leaching for Ni/AC and Ni-Re/AC(n_Ni_:n_Re_ = 1:1) took place after the first run. However, the leaching percentage of Ni for Ni-Re/AC(n_Ni_:n_Re_ = 1:1) (ca. 2.1%) was approximately one fifth that of Ni/AC (ca. 10.2%), clearly demonstrating that the presence of Re evidently enhanced the stability and decreased the Ni leaching. The other factors, such as Ni nanoparticle sintering and poisoning by strongly absorbed degradation products, probably further led to deactivation of the catalyst. Thus, albeit that the Re can substantially improve the catalyst stability and the catalytic performances, further effort needs to be undertaken, which includes the choice of other supports with strong metal–support interaction or other transition metals that may enhance the stability of the Ni based catalyst. 

## 4. Conclusions

A series of Ni-Re/AC bimetallic catalysts with different Ni/Re ratios was developed and used for the hydrogenation of xylose and the xylose containing hydrolysate into xylitol. The bimetallic Ni-Re/AC catalysts exhibited excellent catalytic performances in the hydrogenation of xylose and the hydrolysate from *Camellia oleifera* shell. Ni-Re/AC (n_Ni_:n_Re_ = 1:1) with 5 wt% Ni loading afforded a xylitol yield as high as 98% at 140 °C for 1 h, which was attributable to the synergistic effect between Ni and Re, as well as small Ni particle sizes. The reusability experimental results indicated that the Ni leaching occurred over both monometallic Ni and bimetallic Ni-Re catalyst, but the incorporation of Re remarkably enhanced the catalyst stability and decreased the Ni leaching level during the hydrogenation reaction. 

## Figures and Tables

**Table 1:** Sizes and XPS results of various catalyst.

Entry	Catalyst	Ni Particle Size (nm) ^a^	Ni Dispersion (CO/Ni)
1	Re/AC	10.6 ± 5.0	Nd ^b^
2	Ni-Re/AC (n_Ni_:n_Re_ = 1:1)	13.0 ± 5.0	0.080
3	Ni-Re/AC (n_Ni_:n_Re_ = 2:1)	15.1 ± 6.1	0.027
4	Ni-Re/AC (n_Ni_:n_Re_ = 4:1)	15.8 ± 6.1	/
5	Ni-Re/AC (n_Ni_:n_Re_ = 10:1)	16.0 ± 5.2	/
6	Ni/AC	22.0 ± 6.2	0.020

^a^ The Ni particle size was determined by TEM; ^b^ not detected.

**Table 2:** Effect of different reaction conditions on the hydrogenation of xylose to xylitol ^a^.

Entry	Temperature (°C)	Time (h)	Xylose Conversion (%)	Xylitol Yield (%)
1	120	2.0	79	77.6
2	140	0.5	59	57.2
3	140	1.0	99	98.0
4	140	2.0	99	95.6
5	160	2.0	99	93.0
6	180	2.0	99	85.8
7	200	2.0	99	64.8

^a^ Reaction conditions: 1.0 g xylose, 20 mL H_2_O, 0.1 g Ni-Re/AC (n_Ni_:n_Re_ = 1:1) catalyst.

**Table 3:** Summary of the catalytic hydrogenation of xylose to xylitol with various catalysts.

Catalyst	Tem. (°C)	Time (h)	H_2_ pre. (bar)	Conversion (%)	Selectivity (%)	Ref.
Ru/AC	110	1.5	50	100	98.7	[35]
Rh/perovskite	100	2.0	25	100	51.0	[32]
Ru/polymer	120	2.0	55	99.8	94.1	[34]
Ru/TiO_2_	120	1.0	20	100	98.0	[36]
Ru/Y	120	1.0	55	62	98.0	[29]
NdCeAl_0.162_Ni_0.838_	100	2.0	/	20	50.0	[36]
Raney Ni	120	2.0	55	96.9	96.7	[14]
Ni-Re/AC	140	1.0	20	99	98	This work

## Supplementary Materials

The following are available online at https://www.mdpi.com/2079-4991/10/1/73/s1. Figure S1: TEM images and their corresponding size distributions of (A, A’) Ni-Re/AC (n_Ni_:n_Re_ = 10:1) (B, B’) Ni-Re/AC (n_Ni_:n_Re_ = 4:1) (C, C’) Ni-Re/AC (n_Ni_:n_Re_ = 2:1); Figure S2: Ni 2p XPS of (A) Ni-Re/AC (n_Ni_:n_Re_ = 10:1) (B) Ni-Re/AC (n_Ni_:n_Re_ = 4:1) and (C) Ni-Re/AC (n_Ni_:n_Re_ = 2:1); Figure S3: Re 4f XPS of (A) Ni-Re/AC (n_Ni_:n_Re_ = 10:1) (B) Ni-Re/AC (n_Ni_:n_Re_ = 4:1) and (C) Ni-Re/AC (n_Ni_:n_Re_ = 2:1); Table S1: The hydrolysis reaction of *Camellia oleifera* shell.
