# Supplementary material for: Efficient Hydrogenation of Xylose and Hemicellulosic Hydrolysate to Xylitol over Ni-Re Bimetallic Nanoparticle Catalyst
Source: Nanomaterials (Basel). 2019 Dec 30;10(1):73. doi: 10.3390/nano10010073 (PMC7022744; doi:10.3390/nano10010073)
Supplement: Supplementary file 1 [file nanomaterials-10-00073-s001.pdf]

Supplementary Materials

Efficient hydrogenation of xylose to xylitol over Ni-Re bimetallic nanoparticle catalyst

Haian Xia[1,2]*, Lei Zhang[1,2], Hong Hu[1,2], Songlin Zuo[1,2], Li Yang[1,2]*

[1] Jiangsu Provincial Key Lab for the Chemistry and Utilization of Agro-forest Biomass, College of Chemical Engineering, Nanjing Forestry University, Nanjing 210037, China

[2] Jiangsu Co-Innovation Center of Efficient Processing and Utilization of Forest Resources, Nanjing Forestry University, Nanjing 210037, China

1. TEM results

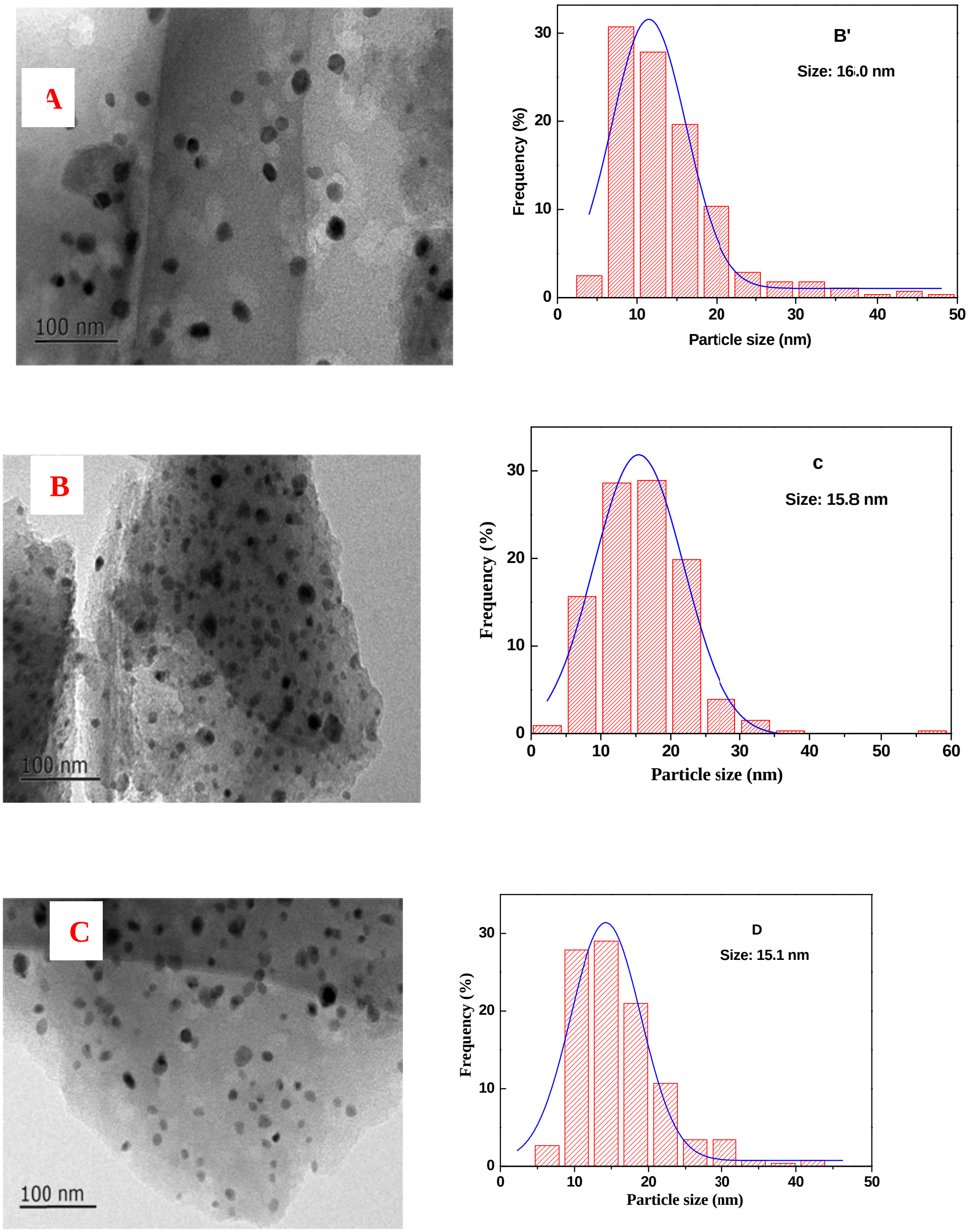

Figure S1 TEM images and their corresponding size distribution of (A, A′) Ni-Re/AC (n_{Ni}:n_{Re} =10:1) (B, B′) Ni-Re/AC (n_{Ni}:n_{Re} =4:1) (C, C′) Ni-Re/AC (n_{Ni}:n_{Re} =2:1)

2. XPS results

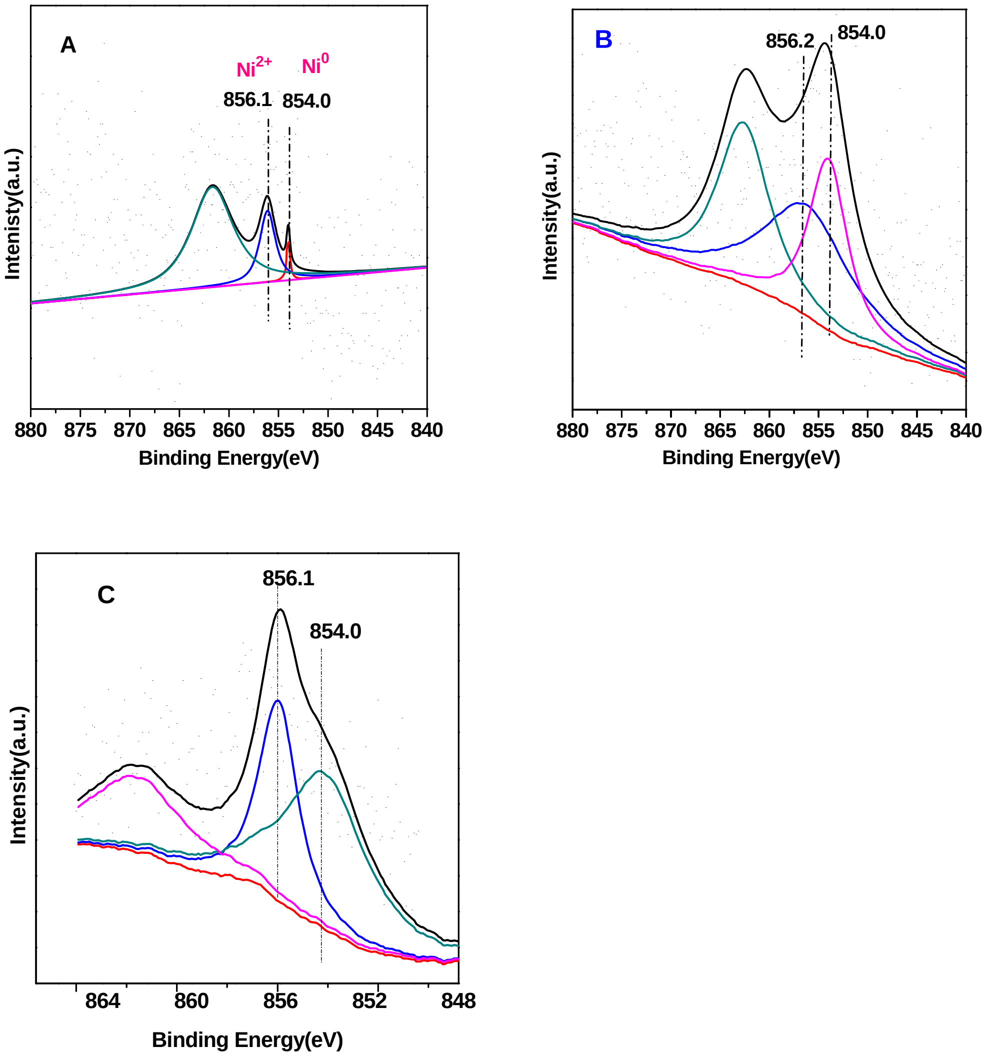

Figure S2 Ni 2p XPS of (A) Ni-Re/AC (n_{Ni}:n_{Re} =10:1) (B) Ni-Re/AC (n_{Ni}:n_{Re} =4:1) and (C) Ni-Re/AC (n_{Ni}:n_{Re} =2:1).

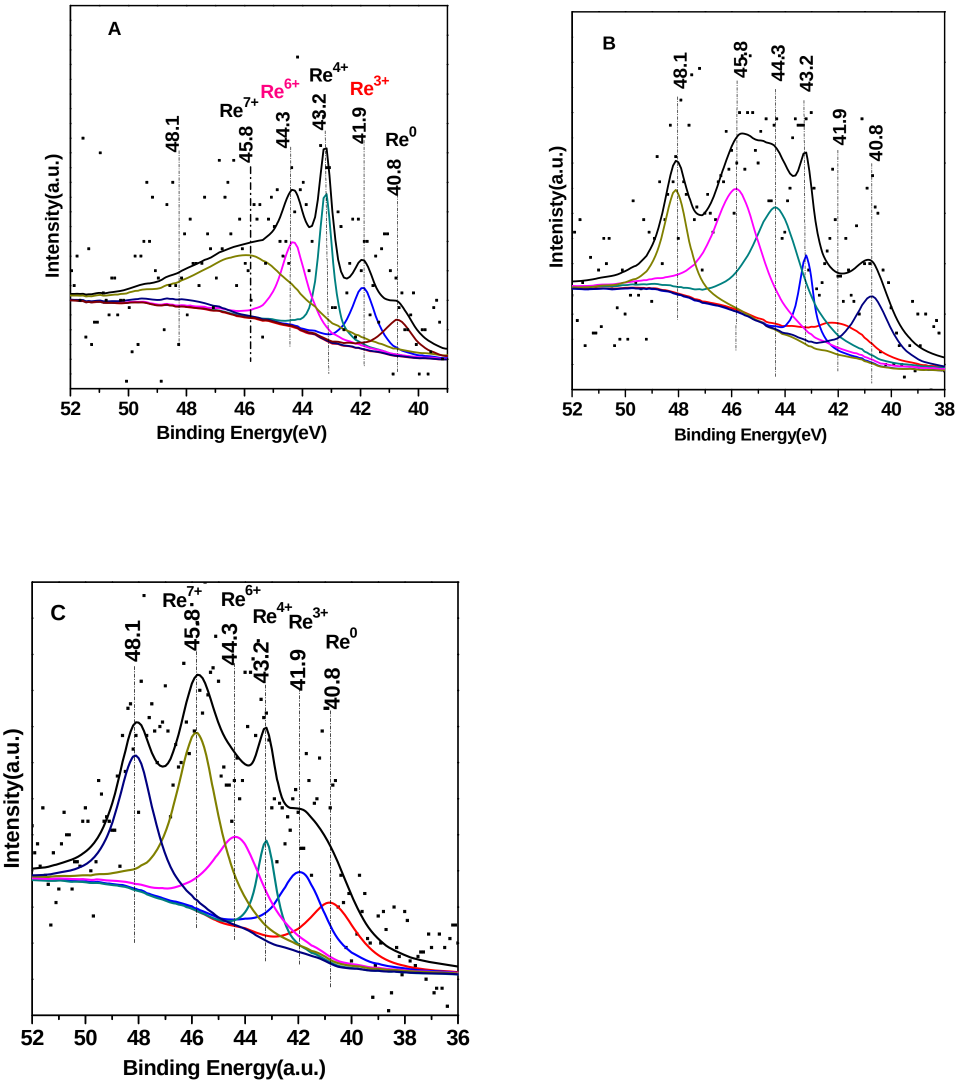

Figure S3 Re 4f XPS of (A) Ni-Re/AC ($n_{Ni}:n_{Re}$ =10:1) (B) Ni-Re/AC ($n_{Ni}:n_{Re}$ =4:1) and (C) Ni-Re/AC ($n_{Ni}:n_{Re}$ =2:1).

3. The hydrolysis reaction of *Camellia oleifera* shell

Table S1 The hydrolysis reaction of *Camellia oleifera* shell

Entry	Temparture(°C)	H_2SO_4 concentration (wt.%)	Time (h)	Xylitol yield (%)
1	60	0.5	0.5	27.8
2	60	1	1	95.1
3	60	1.5	2	96.8
4	80	0.5	1	92.3
5	80	1	2	98.2
6	80	1.5	0.5	96.5
7	100	0.5	2	88.9
8	100	1	0.5	75.7
9	100	1.5	1	63.6
